# Bioactive Titanium Surfaces: Interactions of Eukaryotic and Prokaryotic Cells of Nano Devices Applied to Dental Practice

**DOI:** 10.3390/biomedicines7010012

**Published:** 2019-02-12

**Authors:** Marco Cicciù, Luca Fiorillo, Alan Scott Herford, Salvatore Crimi, Alberto Bianchi, Cesare D’Amico, Luigi Laino, Gabriele Cervino

**Affiliations:** 1 Department of Biomedical and Dental Sciences Morphological and Functional Imaging, Messina University, 98100 Messina, ME, Italy; lucafiorillo@live.it (L.F.); cesaredamico89@gmail.com (C.D.); gcervino@unime.it (G.C.); 2 Department of Maxillofacial Surgery, Loma Linda University, Loma Linda 92354, CA, USA; aherford@llu.edu; 3 Department of Biomedical and Surgical and Biomedical Sciences, Catania University, 95123 Catania, CT, Italy; torecrimi@gmail.com (S.C.); alberto.bianchi@unict.it (A.B.); 4 Department of Experimental Medicine and Surgical Disciplines, Naples University, 80100 Naples, NA, Italy; luigi.laino@unicampania.it

**Keywords:** nanotechnologies, nanomedicine, active surfaces, nanosurface, cells, dental devices

## Abstract

Background: In recent years, many advances have been made in the fields of bioengineering and biotechnology. Many methods have been proposed for the in vitro study of anatomical structures and alloplastic structures. Many steps forward have been made in the field of prosthetics and grafts and one of the most debated problems lies in the biomimetics and biocompatibility of the materials used. The contact surfaces between alloplastic material and fabric are under study, and this has meant that the surfaces were significantly improved. To ensure a good contact surface with the cells of our body and be able to respond to an attack by a biofilm or prevent the formation, this is the true gold standard. In the dental field, the study of the surfaces of contact with the bone tissue of the implants is the most debated, starting from the first concepts of osteointegration. Method: The study searched MEDLINE databases from January 2008 to November 2018. We considered all the studies that talk about nanosurface and the biological response of the latter, considering only avant-garde works in this field. Results: The ultimate aim of this study is to point out all the progress made in the field of bioengineering and biotechnologies about nanosurface. Surface studies allow you to have alloplastic materials that integrate better with our body and allow more predictable rehabilitations. Particularly in the field of dental implantology the study of surfaces has allowed us to make huge steps forward in times of rehabilitation. Overcoming this obstacle linked to the time of osseointegration, however, today the real problem seems to be linked to the “pathologies of these surfaces”, or the possible infiltration, and formation of a biofilm, difficult to eliminate, being the implant surface, inert. Conclusions: The results of the present investigation demonstrated how nanotechnologies contribute substantially to the development of new materials in the biomedical field, being able to perform a large number of tests on the surface to advance research. Thanks to 3D technology and to the reconstructions of both the anatomical structures and eventually the alloplastic structures used in rehabilitation it is possible to consider all the mechanical characteristics too. Recent published papers highlighted how the close interaction between cells and the biomaterial applied to the human body is the main objective in the final integration of the device placed to manage pathologies or for rehabilitation after a surgical tumor is removed.

## 1. Introduction

The nanosurface topic has been debated in recent years due to all the microbiological features and biological interactions with the human body. The new biomaterial nanosurfaces seem to inhibit the formation of biofilms and instead guarantee an excellent integration of “more complex and evolved” cells, like those of the human body organism [1]. The application of surface treatments to the alloplastic materials used in rehabilitative surgery, such as the titanium surface of dental implants, has allowed in recent years to significantly shorten healing times, and in particular to reduce the time required for osteointegration [2]. Within a fashion that provides everything and immediately, the ability to rehabilitate the patient in the shortest possible time is a challenge for clinicians, especially when the patient must heal after a fracture created for implant preparation. In the process of contact osteogenesis there are a series of osteoconductive phenomena, thanks to which the cells endowed with osteogenic potential migrate in the peri-implant space through the matrix constituted by the fibrin network. In the osteogenesis processes, the synthesis of new bone is determined starting from the implant surface in the centrifugal direction. After the deposition of this first layer, strongly influenced by the implant surface, the production and the gathering of the collagen fibers and the bone matrix occurs. The bone subsequently goes into full ripening within four weeks. The following tissue reactions are influenced by both the physical-chemical properties of the material and the topographic features. Initially the surfaces were of the machine type, afterwards they began to treat these last ones, making them nano or micro-rough [3].

The present study analyzes the progress of research in the field of nanotechnology, the characteristics of these surfaces, and the works that could lead to a real revolution in this field, ensuring a lower bacterial adhesion with all the problems that entails. We present a perspective that presents a new point of view on existing problems. Fundamental concepts or prevailing notions on a specific topic explored in this case include proposing and supporting a new hypothesis or discussion of the implications of a new implementation innovation for implant surfaces, thus placing the focus on results and hoping for a revolution in bioengineering.

## 2. Material and Methods

### 2.1. Focus Question

The following focus questions were developed according to the population, intervention, comparison, and outcome (PICO) study design:

- What are the geometric and biological characteristics of a nanosurface?
- What is the interaction between a nano surface and a biofilm? And above all, does this find any difference with respect to microsurface adhesion or other surfaces?

### 2.2. Information Sources

The search strategy incorporated examinations of electronic databases, supplemented by hand searches. A search of four electronic databases, including Ovid MEDLINE, PubMed, EMBASE, and Dentistry and Oral Sciences Source, biomaterials for relevant studies published in the English language from January 2008 to November 2018 was carried out. 

A hand search was also performed in the journals Nanotechnologies and Microbiology. The investigation was limited to English language articles. A hand search of the reference lists in the articles retrieved was carried out to source additional relevant publications and to improve the sensitivity of the search.

### 2.3. Search

The keywords used in the search of the selected electronic databases included the following: “Nanosurface” OR “nanosurface adhesion” OR “nanosurface biofilm”.

The choice of keywords was intended to collect and to record as much relevant data as possible without relying on electronic means alone to refine the search results.

### 2.4. Selection of Studies

A researcher from the University of Messina singularly analyzed the obtaining papers in order to select inclusion and exclusion criteria as follows. Reviewers compared decisions and resolved differences through comparing the manuscripts. For the stage of reviewing of full-text articles, a complete independent dual revision was performed.

### 2.5. Types of Selected Manuscripts

The review included studies published in the English language. Letters and editorials were excluded. 

### 2.6. Types of Studies

The review included all human studies and literature reviews published between January 2008 and November 2018, on nanosurface and biofilm adhesion used for rehabilitative dentistry and implantology.

### 2.7. Inclusion and Exclusion Criteria

The full text of all studies of possible relevance was obtained for assessment against the following inclusion criteria:

- Nanosurface interaction with structures of dental competence.
- Nanosurface of alloplastic structures used in rehabilitative dentistry
- Nanosurface and interactions between bone and implants
- Nanosurface and bacterial adhesion

The applied exclusion criteria for studies were as follows:

- Studies involving patients with specific diseases, immunologic disorders, uncontrolled diabetes mellitus, osteoporosis, or other implant risk related systemic conditions
- Not enough information regarding the selected topic
- Nanosurface not for dentistry or maxillofacial fields
- Articles published prior to 1 January 2008
- No access to the title and abstract in English language

### 2.8. Sequential Search Strategy

After the first literature analysis, all article titles were screened to exclude irrelevant publications, case reports, and no English language publications. Then, research was not selected based on the data obtained from screening the abstracts. The final stage of screening involved reading the full texts to confirm each study’s eligibility, based on the inclusion and exclusion criteria.

### 2.9. Data Extraction

The data were independently extracted from studies in the form of variables, according to the aims and themes of the topic.

### 2.10. Data Collection

Data were collected from the included articles and arranged in the following fields:

- “Author (Year)” – revealed the author and year of publication
- “Type of study” – indicated the type of the study
- “Type of surface” – described the type of surface
- “Surface treatment” – described the surface treatment way

### 2.11. Risk of Bias Assessment

This type of work brings together all the studies in the literature in the last ten years, presenting a technique for nanosurface realization and analyzing their biological interactions. The risk of bias in this case is present because each author was convinced their own study could have influenced the results. For this reason, the works taken into consideration and all those with a high risk of bias or with conflicts of interest have been carefully evaluated.

## 3. Results

### Study Selection

**Figure 1:**
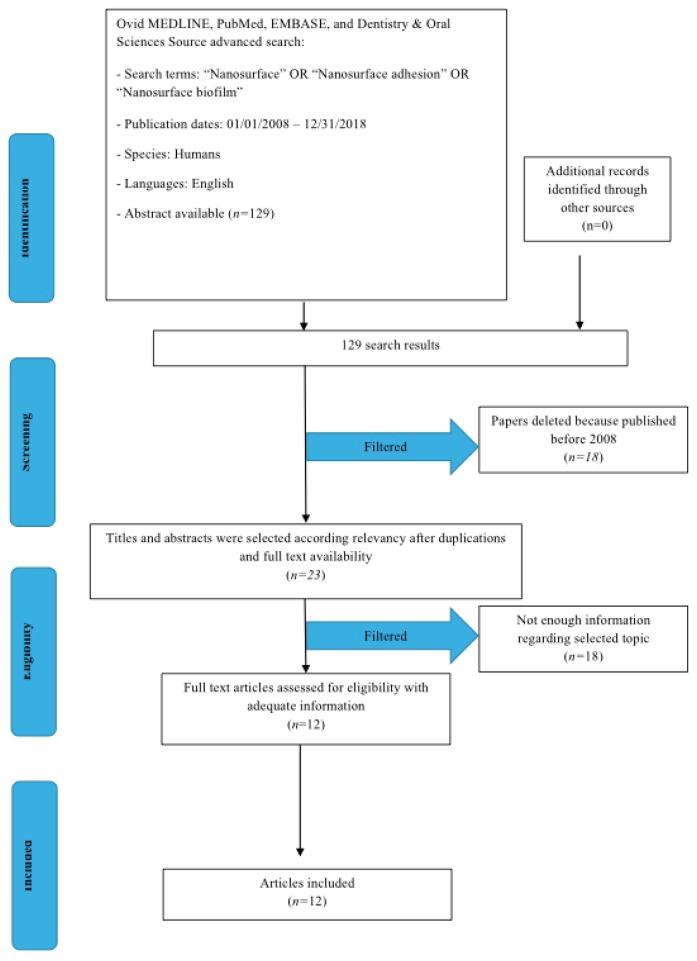
PRISMA flow diagram.

Perspective study and data extraction were performed according to a Preferred Reporting Items for Systematic Reviews and Meta- Analyses (PRISMA) flow diagram (Figure 1). The initial electronic and hand search retrieved 129 citations. 18 papers were excluded because there were published before 1 January 2008. Of 111, 88 were excluded because they were not Free Full Text. At last only 12 studies were included because of the human science field.

## 4. Bioactive Nano Technology and Biomedical Application 

Since 1980, it has been highlighted that there is a different response by the body against different titanium surfaces. The first differentiation was made between smooth and the rough ones. In fact, in the field of orthopedic dentistry or craniofacial surgery, numerous studies have shown greater osseointegration, with a greater Bone-Impact Contact (BIC) of the microrough surface titanium fixtures. It has also been shown as a series of cellular properties and behaviors, such as the expression of certain genes and protein secretions are influenced by the type of surface with which the cells come into contact. Some surfaces improve the adhesion and proliferation of the eukaryotic cells of the body, however, they also allow for greater adhesion by bacterial cells and fungi. Precisely in this case surfaces with a nanopatterning come in day, as these have a lower proliferation and adhesion by prokaryotic cells, are more rudimentary, and are not able to interface with a nanometric surface.

### 4.1. Dental Implants Surfaces

There are several publications that demonstrate the importance of the characteristics of the implant surface to achieving a better result and specifically it has been seen how the surface microscopical features can influence the behavior of the cells responsible of the final bone formation. In blasted implants, after four weeks, mature bone appears to have direct contact with the implant surface, but in many areas the interposed osteoid matrix is not yet mineralized. In other areas of the implant perimeter it is possible to observe the formation of an osteoid matrix directly on the implant surface. Bone formation at the bone-implant interface is a process that involves cell migration and proliferation, followed by synthesis, deposition, and mineralization of the bone matrix [3]. All these phases involving the neoapposition of bone around the implants can be influenced by surface roughness, even if a change in surface morphology can also influence surface energy and chemical composition. The optimal surface roughness has not yet been defined with certainty, although it has been suggested that a roughness of 1.5 μm tended to reduce the time of a bone response compared to those observed around surfaces with lower or greater roughness. It is known that by changing the geometry and the microporosity of a biomaterial it is possible to make it more osteoconductive or even osteoinductive. Surface roughness affects the wettability of the biomaterial in the water and protein content present in the implant site, making it possible to absorb BMP (morphogenetic) proteins, OP (osteogenetic proteins), fibronectin and osteopontine released at the implant site following surgery. It is clear how the composition, roughness, topography and surface energy play an important role during the initial phases of implant integration. It is widely demonstrated that sandblasted implants have shown higher percentages of bone-to-implant contact than smooth, machined implants. Surface roughness causes an increase in osteoblast proliferation and preosteoblastic cell differentiation, as well as migration to the implant site and an increase in the production of alkaline phosphatase, transforming growth factor (TGF) beta, prostaglandin 2 (PGE2). Alkaline phosphatase plays a fundamental role in the mineralization processes of the osteoid matrix and of the immature bone, while TGF beta and PGE2 have the function of stimulating the preosteoblastic cells to differentiate and migrate to the implant surface. Osteoblasts grown on more rough surfaces showed an increased matrix production and an increased expression of alkaline phosphatase. The literature is rich in comparisons between plasma-sprayed titanium implants (Titanium Plasma-Sprayed, TPS) and sandblasted and etched titanium (Sandblasted Large-grid and Acid-etched, SLA).

In temporal terms, considering the above experimental confirmations of the new SLA reality, and complying with the moments of physiological bone healing, it will probably be possible to accelerate this phase from 12 weeks up to a value of 6 weeks, as indicated by the encouraging results of an ongoing study. For sandblasted and etched surfaces, sectional examinations performed during the integration process showed that at 7 days the osteoid matrix is predominantly non-mineralized. The percentage of bone-implant contact is about 14.5%. At 14 days between the turns of the implant there are numerous areas of new bone formation; in addition, osteoblasts secreting new osteoid matrix are present. No multinucleated cells or inflammatory cells with pathological significance have been found. The percentage of bone-implant contact is 21.67%. At 30 days it is possible to observe an intimate contact between bone and implant, as no gaps are presented at the interface nor is fibrous connective tissue interposed. The new bone formation occurs above all towards the implant surface. The percentage of bone-implant contact is about 34.5%. At 60 days the osteoblastic activity is poor and, only in some fields, some osteoblasts secreting the osteoid matrix are observed. No multinucleated cells or cells typical of acute or chronic inflammation are observed. The percentage of bone-implant contact is 58.83%. The percentage of contact between bone and implant at the interface level (BIC-Bone to Implant Contact) and the superficial morphology of the implant are crucial factors for the long-term success of osseointegrated implants [1,2,3,4]. The resistance of screw systems increases in relation to BIC, underlining the importance of this parameter. The microtopography of the implant surface is able to influence BIC and cellular activity and therefore influence the long-term survival of osseointegrated implants. Implants with a rough surface are able to obtain a better bone anchorage compared to smooth or machined implants, as they obtain a much higher percentage of BIC. In order to obtain a superficial topography able to favor the osseointegration process, different surface treatments of the implants have been introduced, such as sandblasting, etching and hydroxyapatite coatings [4,5,6].

### 4.2. Surface Treatments

Surface treatments of metals are intended to produce a biologically active surface. Already from a macrosurface, geometric point of view, retention increases in the tissues being formed. In order to obtain an ideal, efficient roughness as already demonstrated, different techniques have been proposed.

The implant surfaces are subdivided into two large groups, the smooth and the rough ones. Rough surfaces can be obtained with two types of treatments, additive and subtractive techniques.

Additive techniques:

- Titanium Plasma Spray
- Coating with hydroxyapatite
- Anodic oxidation

Subtractive techniques:

- Sandblasting with alumina oxide
- Sandblasting with titanium particles
- Sandblasting with soluble or reabsorbable materials
- Etching with strong acids
- Double acid etching

It is also possible to find new combined techniques that involve sanding and acid etching or sandblasting and thermal etching. The smooth implants can be electropolished or machined, the former having a surface that is subjected to an electrochemical treatment by immersion in electrolytic solution. The implants with a machined surface have a surface that appears shiny and smooth but in any case show streaks. Other surfaces are those (TPS) with the treatment of Plasma surface, therefore titanium powders. The problem with this technique is the bad control of contamination and the possibility of the detachment of particles from the metal surface. There are also surfaces covered with hydroxyapatite, the latter binds to the patient’s bone and does not induce toxic or inflammatory phenomena. The sandblasted and etched surfaces defined as SLA are surfaces with coarse-grained and acid-etched sand. SLA surfaces have a larger contact surface than the roughest Plasma-Spray. There are also surfaces coated with biologically active glass, experimentation on these surfaces has shown positive characteristics. The glass material is against resorption and degradation with complete replacement by the bone tissue. These surfaces are characterized by a high wettability. In any case, all the surfaces before marketing are sterilized with treatments that do not affect the characteristics of the metal. The purity of the surfaces and the absence of contaminants is a much debated element that influences the quality and the cost of the material itself [6].

#### 4.2.1. Nanotecnologies and Their Relation on Macro Biomaterial 

This type of configuration is produced following an electrochemical anodizing process, which is innovative for the creation of a nanostructured morphology, which is finely characterized. The implications of these surfaces have only recently begun to study animal models. The porous structure allows, with the anodic process, to deposit on the surface of the phosphates with osteoconductive power; anodized implants show good performance compared to sandblastes. In the latter the response in peri-implant soft tissues is good from the early stages of osteogenesis, but in nanoporous there is certainly a greater bioactivity, and a higher percentage of bone-to-implant contact. At the end of the process there is a bone formation in both, but in nanoporosis the new tissue is also characterized by better continuity. The advantages of these surfaces are not only attributed to the roughness given by the pores, which in many cases is also similar as values to other types of surfaces, but above all to the chemical influence of the anodized layer. The anodized nanoporous surface offers good mechanical retention and the pores are controlled in size to allow efficient transport of factors and proteins useful for the osseointegration process. This type of surface therefore allows the proliferation of osteoblastic cells, but also of connective tissue cells and epithelial cells, having instead a negative activity in the formation of a biofilm [7,8].

The possibility to realize a biomaterial working on its nanostructure gives both the clinicians and researchers a unique set of opportunities. The usual way to create tissue engineered implants relies on providing a desired element homogeneously throughout a biomaterial, which is related to top-down tissue engineering [1,2,3,4,5,6,7,8].

Specifically, an implant’s integration in the human body is ruled by its ability to provide protection from the implantation site’s often-harsh environment, facilitating host-implant integration by, e.g., attracting blood vessels and offerring continued stimulation to guarantee long-term tissue function.

As for the bone substitutes applied to orthopedic or cranio-maxillofacial surgery, the biomaterial used in the place of the bone graft should have clinical features similar to autologous bone, as per consistency and quantity. However, the micro features of the materials replacing the human bone nanostructures are fundamental in order to achieve the final graft integration [9,10].

During the last years, research has been performed in the development of distinct nano- and micromaterials using several methods. However, a key determinant of success is grounded in the efficient, elegant, and cost-effective integration into clinically relevant therapies. Indeed, the integration of nano- and microtechnologies in bottom-up tissue engineering approaches represent a unique opportunity to make these elusive man-made hierarchical tissues a reality [11,12,13].

#### 4.2.2. Biofilm and Bacterial Adhesion on Surfaces

A biofilm is a complex aggregation of microorganisms distinguished by the secretion of an adhesive and protective matrix. The formation of a biofilm begins with the anchoring of microorganisms freely floating to a surface. The first “settlers” adhere to the surface initially through weak and reversible Van der Waals forces. Biofilms allow the survival of bacterial cells in a hostile environment. If the colonizing bacteria are not immediately separated from the surface, they can anchor more stably using cell adhesion molecules, such as pili. Early colonizers facilitate the arrival of other cells by providing several sites of cell adhesion and begin to build the matrix that allows the integrity of the biofilm. Some species are unable to attach themselves to a surface autonomously, but often manage to anchor themselves to the previous matrix or colonizers. Once the colonization has begun the biofilm grows through cell division and integration of external bacteria, including other species. Dental plaque is a biofilm produced by the bacteria present in the oral cavity as Streptococcus mutans. Biofilms can grow on the surface of solid implants in the body. Biofilms that propagate along implanted tubes or wires can generate pernicious infections in patients. For example, Staphylococcus epidermidis can produce biofilms on venous catheters [13,14].

#### 4.2.3. Dental Implants and Perimplantitis

The dental implant is a medical device of a surgical type used to functionally and aesthetically rehabilitate the congenital loss or lack of one or more teeth, allowing the support of a prosthetic substitute through direct bone support thanks to a biological process known as osseointegration. It can be inserted into both the upper or lower maxilla. The implant element is inserted immediately, so that osseointegration occurs more easily, while only afterwards the visible dental prosthesis is added; a variable amount of time is therefore necessary to achieve correct osseointegration. During the physiological bone remodeling, the complex submicronic three-dimensional structure produced by the action of the osteoclasts represents the substrate on which the so-called cement line (grafting line) is constituted by a collagenous non-collagenous matrix deposited by the osteoblasts. The interdigitation of these two structures is of fundamental importance and strictly depends on the submicronic surface topography of the residual matrix. For this reason, for biomimetic purposes, the modern implant surfaces have to reproduce the structural characteristics of the residual matrix and therefore be nanostructured. A material can be defined as nanostructured if it has constituents smaller than 100 nm. The human bone can be considered a clear example of a complex material with nano-structural characteristics: in fact, the non-collagenic proteins of the matrix, together with the collagen fibrils and HA crystals, which are natural constituents of the bone, are nanometric structures; in the same way, the osteons, the haversian systems and the bony lamellae are structures of micrometric dimensions, while the spongy bone and the cortical bone as a whole represent superior structures of millimetric dimensions. Nanostructured surfaces seem capable of modifying the tissue response. First of all, they have an extensive surface area and high surface free energy and wettability, able to attract and bind an extraordinary quantity of proteins. Just the formation of a biofilm and the bacterial proliferation on the surfaces makes it unpredictable, and from an expiration date to our implant rehabilitations. Therefore, it should be considered that in the oral environment, in addition to the multitude of bacteria and fungi, there are a whole series of chemical substances, also related to food, which can alter the surface of our fixtures, especially if these are already exposed to inflammatory reasons or other. The development of a feared pathology, defined as perimplantitis, which represents the new challenge of dentistry, can lead to the loss of our oral rehabilitation [15,16] as we can observe in Image 1. There are techniques of periodontology to intervene and limit the damage, but obtaining a re-osteintegration is an argument still debated. Surely bone regenerations, also using materials of different derivation [17], are to be excluded on an inflamed site. Inflammation is self-sustaining thanks to the presence of a whole series of biomolecular factors and therefore linked to inflammattory mediators [18]; but also for other causes of a completely different kind, such as the mechanical stress and torsion of our fixtures or implant components that can further cause damage to the bone tissue and to the peri-implant soft tissues [19,20]. 

## 5. Discussion

**Figure 2:**
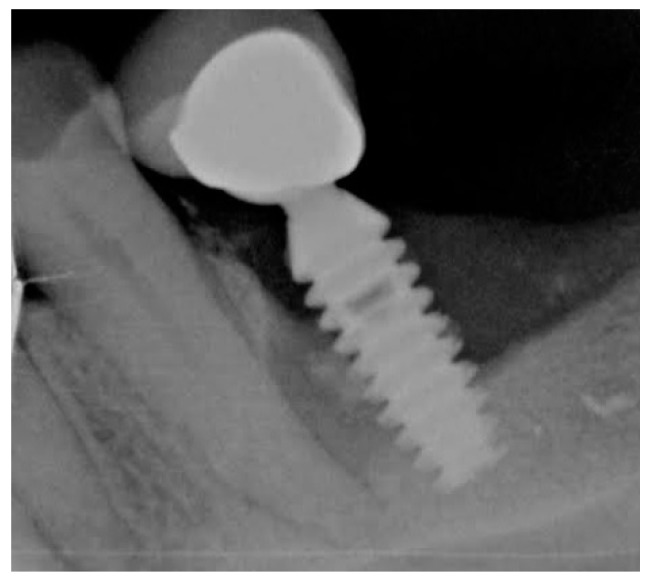
Image obtained during clinical practice of Dr. L. Fiorillo. Radiographic examination of implant with peri-implantitis due to the formation and organization of a biofilm on the implant surface. The interface between the bone tissue and the implant has been interrupted, this must be minimal and in the absence of biofilm to guarantee integration.

**Figure 3:**
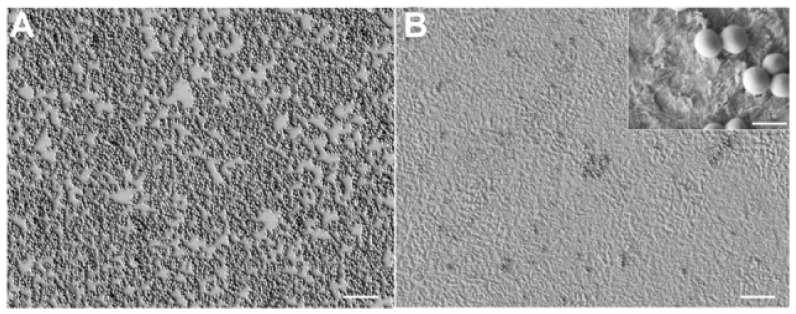
Adapted from [1] with permission of Prof. A. Nanci and Dove Press in 2018. Biofilm adhesion on different surfaces: (**A**) dental implant smooth surface; (**B**) dental implant mesoporous surface on the right.

**Figure 4:**
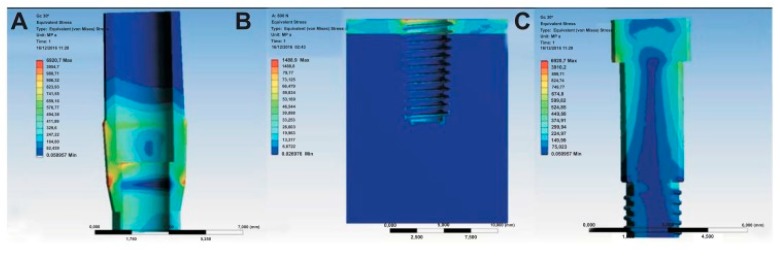
Adapted from [24] with permission of M. Cicciù in 2018. Von Mises and Stress distribution on three different prosthodontics sections; (**A**) located in the abutment section; (**B**) stress located at the bone tissue; (**C**) stress distribution at the passant screw.

Dhawan et al. in 2016 conducted a study about cell-nanosurface interaction for implant improvement. In this study they used tantalum oxide nanodots of 50 and 100 nm diameter with an interdot of 20–70 nm. This promoted cell viability and spreading area, upregulating transcription factors and genes responsible for bone protein secretion too. This study found that nanotopographic features can vitally control the cell and should therefore be taken into account when designing implants [21]. Boyan in 2016 studied the interaction between implant surfaces and mesenchymal stem cell differentiation and maturation. In this study authors investigated integrins (transmembrane receptors) that recognize changes in the surface and mediate signaling pathways. Integrins can be implicated in osteoblastic differentiation of cells on titanium surfaces [22]. Implant surface modification of titanium can induce bio-mimetic features. Zhu tried to improve human bone marrow mesenchymal stem cell attachments and differentiation on implant treated surface. This treatment, called, plasma treatment, can be an ideal method of integrating titanium with native bone for orthopedic applications [23]. The nanostructured magnesium had effect on osteoblast function on a Weng 2013 study. The nanoscale surface of magnesium increases bone formation, compared with normal magnesium not nanostructured [24]. According to Variola in 2011, a nanoscale surface modification can be medically relevant on metals. The nanoscale surface properties stimulate and guide various molecular and biological processes at the implant issue surface [25]. Some methods for making nanostructured surfaces like oxidative patterning, can modulate the expression of cell activity and stem cells. This phenomenon can lead to a new generation of intelligent surfaces that can control the biological response at the site of healing [26,27]. These surfaces according to other studies can generate a nanopatterning surface with antimicrobial properties. Variola et al., in a study coordinated by Antonio Nanci in 2014, evaluated the adherence of *Staphylococcus aureus*, *Escherichia coli*, and *Candida albicans* into titanium disks with mesoporous and polished surfaces compared. A simple chemical oxidative treatment that generated a nanotextured surfaces with antimicrobial capacity with potential application on dental implants industries. The treatment of titanium with an economic mixture of H_2_SO_4_/H_2_O_2_ generates a predictable mesoporous titanium surface layer comprised of a network of nanopores [1]. The problem at this point is about correlation by surface modifications and the biomechanical properties of implants [28,29]. The mechanical characteristics of implants and how to position them, tilt them, and connect them to the prosthesis have been largely studied [30,31,32,33,34,35]. Another technique is represented by an implant titanium surface covered with metal foams. The Ti foam coating is biocompatible on animals and it sustains bone formation and can potentially improve osteointegration [36]. Wazen evaluated the gene expression profiling during the healing phases. Gene expression around nanotextured implants differed from that around the machined-surface implants and empty holes; increasing BIC [37]. The scanning microscopy is a further technique proposed to evaluate the implant surface and also other oral tissues. This clarifies the adhesion of a bacterial biofilm on the implant surface and highlights the dynamics of biofilm formation on these surfaces (Figure 2, Figure 3 and Figure 4) [38,39]. 

### Limitations

This type of analysis does not want to be a review of the articles taken into consideration, but is a way to enlighten the scientific population about the presence of these works, and about the potential of these surfaces on different fields, such as orthopedics, surgery in different bodily districts, and implantology. The study offers a valid alternative and a way to follow for implant houses that still shyly rely on this type of surface, which can also paradoxically be obtained in a economical way.

## 6. Conclusions

Recent research in the field of tissue engineering has investigated new nanotechnologies in order to offer clinicians and patients a better management of the field of regenerative medicine. Specifically, the titanium surfaces applied to orthopedic or craniofacial surgery have been investigated in order to achieve quick integration in the case of bone reconstruction surgeries. The nanotechnology gives significant results when working on surfaces able to attract cells favoring the healing and integration process in the human body. Some published papers defined these new surfaces as intelligent, or a selective-microbial surface. Thus, in the field of orthopedics or oral implant surgery, it is widely considered an incredible advantage. Starting to exploit the potential of these surfaces, which in many cases can be produced with simple and inexpensive methods, is the first step towards the success for this type of rehabilitation. Having a dental implant, which is also in contact with the oral cavity, does not favor the formation of a biofilm and remains unscathed, which can only be an advantage. Indeed, at this point and only at this point could it create the ideal conditions for re-osteointegration. It would be interesting at this point to evaluate the presence of alterations of the mechanical strength of the same fixtures in the case of a total treatment with a nanosurface and to evaluate the results in vivo, which according to this analysis seems to be nothing but promising.
